# Dr. W. H. Dwinelle

**Published:** 1852-07

**Authors:** 


*Dr. W. H. Dwinelle,* formerly one of the editors of this Journal, and known to our readers as an able writer, will start for Europe on the 28th of this month. He will go out in the Asia, and during his absence, will communicate for the Journal everything likely to be interesting to the profession, which may come under his observation while abroad. We wish him a pleasant trip, and a safe and speedy return.

